# Effects of a reimbursement change and travel times on the delivery of private and public radiology services in Norway: a register-based longitudinal study of Norwegian claims data

**DOI:** 10.1186/s12962-019-0190-7

**Published:** 2019-10-16

**Authors:** Anastasia Mokienko

**Affiliations:** Department of Health Management and Health Economics, University of Oslo, P.O. Box 1089, Blindern, 0317 Oslo, Norway

**Keywords:** Radiology, Diagnostic imaging, Reimbursement mechanisms, Fee-for-service plans, Outpatient Health Services, Delivery of Health Care, Supply and distribution

## Abstract

**Background:**

The variation in the impact of the 2008 reimbursement change for Norwegian radiology providers, depending on the travel times to private and public providers in different municipalities, was examined. The activity-based fund allocation for radiology providers was reduced from approximately 50% to 40%, which was compensated by an increased basic grant. The hypothesis was that the Norwegian population would be affected by the reimbursement change unevenly depending on their distances to different types of the providers.

**Methods:**

The study of the effect of the reimbursement change and travel time difference between private and public radiology providers in Norway (*Time_difference*) on the number of the services was performed using fixed-effects regressions applied to panel data at the municipality level with monthly observations for the period 2007–2010.

**Results:**

After the reimbursement change, the number of private services decreased more than the number of public services. Private services declined after 2008, but the absolute value of the effect was smaller as the *Time_difference* became greater. The number of public services increased as the *Time_difference* grew. The total number of services decreased until the *Time_difference* was equal to 40 min and increased for time differences greater than 40 min.

**Conclusions:**

The messages for policymakers are as follows. Populations that only had private providers nearby were more affected by the reimbursement change in terms of a reduced number of services. The reimbursement change contributed to the reallocation of patients from private to public providers. The difference between the centralities of municipalities in their consumption patterns was reduced and the difference between different Regional Health Authorities was increased due to the reimbursement change.

## Background

Radiology services are useful tools in the diagnostic process. When physicians suspect a particular diagnosis, they often send patients for further examination. Sometimes, a diagnosis is confirmed and sometimes not. The question is if we can say that the service was unnecessary if the diagnosis was not confirmed? Some policymakers would say yes. However, a negative answer is still relevant for diagnostics. A debate exists about efficiency, cost savings, and cutting unnecessary services. An extensive amount of literature supports the idea that rising costs do not necessarily translate into an increase in the quality and efficiency of health care [1–5]. However, it is difficult to assess the benefit of diagnostics and specialist services, which makes it easy to say that those services are overused [6]. Diagnostics may save a significant amount of budgeted money by helping physicians more precisely assess diagnoses. For example, a study analysing radiology services at Glasgow Commonwealth Games indicates that imaging played a vital role in providing quick and effective management of injured athletes by delivering quick diagnostics [7].

However, when the benefit of diagnostics is unclear, physician discretion is involved, and practice variation should be expected. This variation could result in patients being overtreated or undertreated. There is evidence indicating some patterns of overutilization. For example, a study from Serbia analysing data from 2007 to 2010 confirms irrational prescribing of diagnostics procedures and necessities of cutting costs [8, 9]. In addition, patterns of overutilization are supported by evidence from the USA, where authors found that prior-authorization slowed growth in the utilization of MRI and CT [10]. Another study from the USA, however, indicated that diagnostic imaging services are being scrutinized and their associated reimbursement has been greatly reduced in the USA partly because they become less visible in and isolated from the clinical arena. However, engagement of radiologists in patient-care teams is essential for patient care decisions [11].

In addition to variations in physician judgement, regional differences in utilization rate might be explained by heterogeneous distribution of services [12]. Several studies from Italy, the USA and Norway on the accessibility of medical service providers have demonstrated that greater travel distances to providers lead to reduced utilization of health-care services [12–16]. Evidence from Australia suggests that there is also a difference in the geographical tolerance of highly versus sparsely populated communities: Residents of closely settled areas are much less willing to travel to access a general practitioner (GP) than people in sparsely populated areas [17]. Thus, people who live in remote areas travel to health-care institutions less frequently than those in populated areas, but they are willing to travel much longer distances than people residing in population centres.

Variations in the provision of health care services can also occur due to differences between public and private providers and their geographical distribution. A study from Brazil indicates that for acute appendicitis, the waiting time for surgery in public hospitals was longer than in private hospitals [18]. In Australia, public and private hospital use is interchangeable: patients often receive diagnostics at private hospitals and then are transferred to public hospitals for operations and checked again at private hospitals [19]. A review study by Basu et al. who analysed 102 articles on private and public health care in low- and middle-class countries, found that reported efficiency tended to be lower in private than in public sector. Private providers have poorer patient outcomes but greater reported timeliness and patient hospitality, while the public sector has more limited availability of equipment, medication, and trained health care workers [20].

Another review article by Tynkkynen and Vrangbæk, of 17 studies representing more than 5500 hospitals across Europe, indicates that public hospitals are most frequently reported as having the best economic performance compared to private for-profit and private non-profit hospitals. However, a sizable number of studies do not find any significant differences between these hospital types. The results in terms of quality are mixed, but results on patient selection indicate public hospitals more often treat older patients, patients with lower socio-economic status, and patients with higher levels of comorbidity and complications than do private hospitals [21].

Several studies indicated geographical variation in consumption of radiology services in Norway. Nevertheless, not all studies have connected the variation to coexistence of private and public providers. For instance, Søreide et al. found regional differences in performed distal pancreatectomy covered by National Health Insurance in Norway. Regional variation persisted for age-and-gender-adjusted rates [22]. A study by Gransjøen et al. (2018) analysed diagnostic imaging of the musculoskeletal system from 2016 and found geographical variation, especially for ultrasound and CT, MRI of the shoulder and radiography of lower back and shoulder, which might indicate overuse or underuse of services [23]. Lysdahl and Børrentzen, who analysed survey data from 2002, found substantial variation especially for CT and MRI. They say that a likely cause is accessibility and coexistence of private and public providers [24]. Our study researches this topic deeper and contributes by connecting the regional variation to differences in the reimbursement settings between private and public providers and to travel times to these providers.

Equal access to good quality care is one of the top priorities of health care in Norway [25, 26]. Understanding the variation in the provision of health care services and how this variation affects political and financial changes helps policymakers make more thorough decisions. To reach this goal, evidence on the contributing factors for regional differences is needed. The aim of this paper is to provide more evidence on what may add to regional differences in provision of health services, using the example of the radiology services in Norway. The study examines how the change in the remuneration system in 2008 for radiology providers contributed to a change in the radiology supply in the different geographical regions, depending on the travel times to private and public providers.

This topic is important because Norway is a vast country with a small population, and it therefore has many remote municipalities. Not all municipalities have radiology providers, and from some municipalities, the travel time can reach several hours. Some municipalities (medium and large) have private and/or public providers, but others do not. Private and public providers reacting differently to financial changes could result in a variation in supply to patients who have a particular kind of provider available.

## Methods

### Public and private providers

Norway has four regional health authorities (RHAs) named after their locations (Southeast, Central, North, and West). There are two types of radiology providers in Norway: private and public. Private providers operate as for-profit institutions that can have contracts with RHAs and deliver radiology services on public terms (Patients only pay the laboratory a patient co-payment, while the rest is paid by the state and the RHA.). Each RHA chooses a number of private radiology providers through a tendering process and by signing contracts with them for a specific number of services. This option is sometimes associated with wait times for patients. Private providers also deliver radiology services on private terms (when patients pay the full fee directly to the laboratory); this option is not associated with patient wait times.

The contracts with RHAs specify the volume of and reimbursement for examinations, the maximum number of services, and the total costs. Some contracts specify only an aggregated budget for services [27]. Other contracts are detailed and specify the budget for each type of service, such as ultrasound imaging (UI), magnetic resonance imaging (MRI), computed body tomography (CBT or CAT scans), and radiography (X-rays) [28].

Public providers are hospital radiology departments that deliver radiology services to the population on public terms; that is, they accept both patients from hospitals and outpatients referred to them by GPs and specialists. Visits to a public or private laboratory require a referral from a GP or a specialist to be covered by the National Health Insurance (NHI) [29]. In theory, radiology laboratories can decline to make an appointment, but in practice, this does not happen often because GPs already act as gatekeepers [30].

### The 2008 reimbursement change

Reforms in the financing of specialist health care have been carried out since 1997 and activity-based funding (ABF) was introduced to encourage the achievement of activity targets ([31], p. 69). If these targets were not met, the RHAs lost income. If the activity levels were higher than targeted, then the costs would be only partially compensated. Hence, ABF was not intended to cover marginal costs or to encourage activity beyond the target ([32], p. 13).

**Fig. 1:**
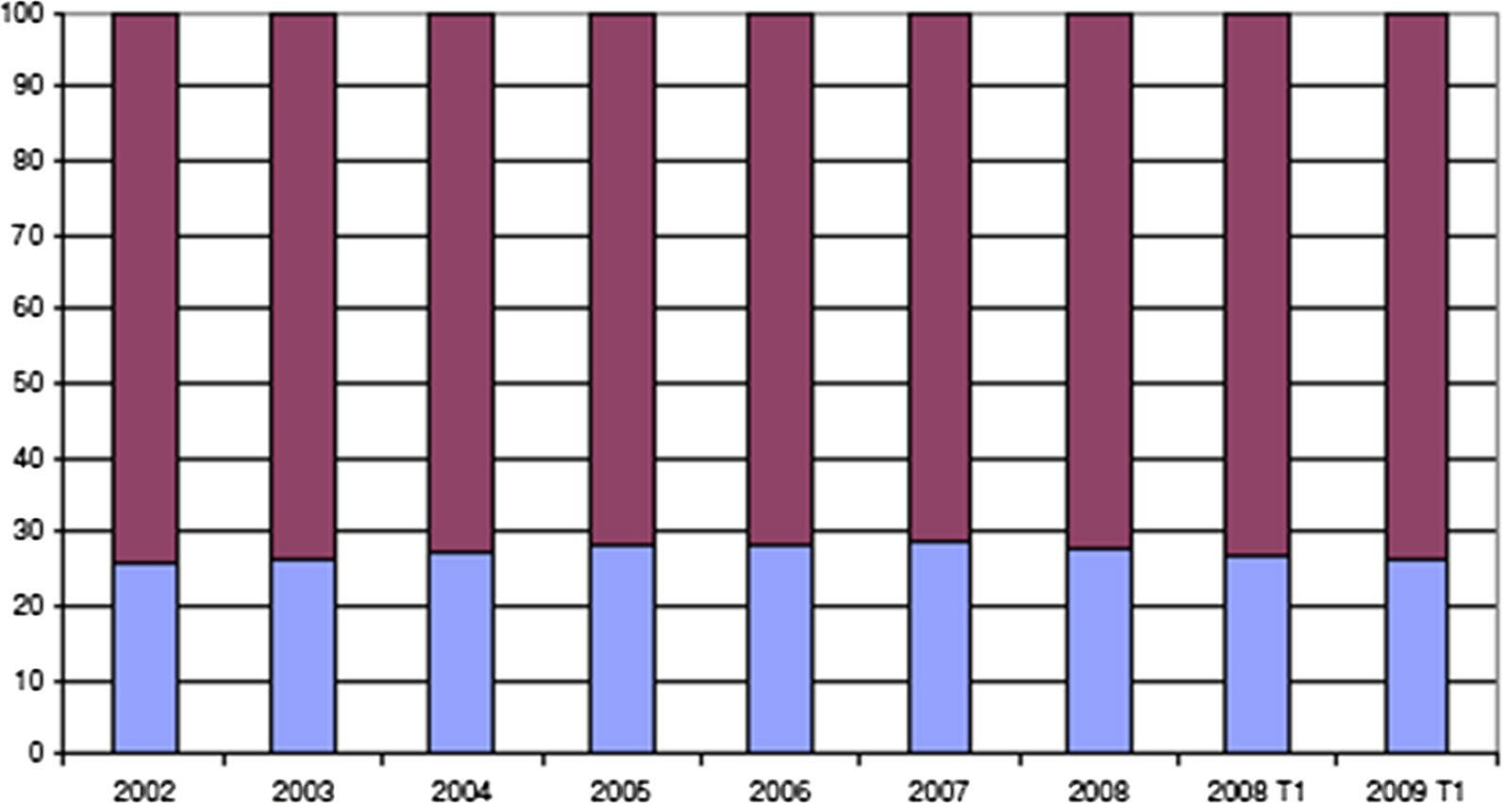
Market share in the costs between private (blue) and public (red) radiology providers in percent. On the Y-axis: percent of market share in the costs between private and public radiology providers. On the X-axis: years, T1—first tertial of corresponding year (Figure 3.4 in [33]. Permission to use Figure 19/284679-1 by Norwegian Health Economics Administration)

For radiology services, ABF funding was first introduced 1 September 2005 to encourage RHA to take more responsibility for planning and prioritizing provision of radiology services [32–34], (p. 248 in [35]). Between 2005 and 2008, the proportions of activity-based and basic allocation were approximately equal. Figure 1 demonstrates that, prior to 2008, spending for private radiology continually increased.

The reimbursement change of 2008 changed radiology funding accordingly: from 1 January 2008 the ABF part decreased from 50 to 40%, and the basic allocation increased from 50 to 60% (from RHA) to compensate. The reimbursement scheme was changed to cut spending and to harmonise the financial scheme of radiology providers with the general system for financing outpatient medical services in Norway [32, 36]: ‘The aim was both to contain costs and to give providers sufficient flexibility to assure the best mix of services for patients’ [31].

In practice, introducing ABF meant that private providers had to enter into agreements with RHA to produce the agreed number of services and receive refunds. These providers would still receive ABF from NHI and co-payments from patients if they produced more services than agreed with RHA. The providers’ revenue thus included three components: the fee-for-service from the NHI scheme (or ABF), patient co-payments (the same for both private and public providers when received through NHI), and the invariable component (a basic allocation independent of the number of the services provided). The size of ABF was based on diagnostic related groups [31], while the size of the basic allocation was decided by several factors, including the number of inhabitants living in the region and the demographics of the population [32].

The public providers’ revenue also included three components equivalent to those of the private providers, but with a different reimbursement mechanism. The RHA and NHI did not reimburse public radiology laboratories directly; instead, they reimbursed the hospital affiliated with the laboratory. Thus, public outpatient providers were not as restricted by contracts as their private counterparts, so they had softer budget constraints than private providers did. Soft budget constraints are often related to a poor ability to balance budgets and providers with the tendency to increase activity or costs to a level above that preferred by the principle stakeholder [37–39]. In contrast, in the private sector, the number of services was controlled by hard budget constraints to maintain positive profits because contracts included specified volumes.

### Data

Claims data were obtained from the Norwegian Directorate of Health. The dataset (aggregated at the municipality level) contained the number of radiology services (CAT scans, MRIs, X-rays, and ultrasounds) reimbursed per month by NHI from 2007 to 2010, the travel times from the patient’s municipality to the municipality with closest private or public provider, the number of inhabitants, the centrality of the municipalities, and the RHAs to which they belonged. Thus, 422 municipalities in 48 different periods (monthly observations from 2007 to 2010) were monitored for a total of 19,867 observations.

### Variables

#### Travel times

Table 1 in Appendix contains an overview of the variables. The travel times were measured in hours according to driving time by car (provided by Info Map Norway [40]) between a patient’s residential municipality (approximated by the municipality of the patient’s GP) and the municipality of the public radiology provider (*Pubtime*) or the private radiology provider (*Privtime*). If patients had a radiology provider in their own municipality, then the travel time was set to zero by definition in the dataset. The difference in travel time between the nearest private provider and nearest public provider is represented by *Time_difference* = *Privtime − Pubtime*. The difference in travel time is included as the main independent variable because, when deciding between two providers in the settings of unevenly distributed providers, patients often choose a closer provider. Since private and public providers have different institutional settings, this choice affects the outcome.

#### Centrality/municipality level

Statistics Norway classifies every municipality in Norway by centrality. During the observation period, centralities were 0A and 0B, 1A and 1B, 2A and 2B and 3 [41]. In the data set, 0A and 0B is denoted by *Centrality0*, 1A and 1B by *Centrality1*, 2A and 2B by ‘*Centrality2’*, 3 by *Centrality3* (*Centrality0* through *3* are dummy variables), where *Centrality3* represents the most central type of municipality (e.g., Oslo), and *Centrality0* denotes the least central ones (e.g., small remote villages).

Centrality indicates the location of municipalities in relation to urban settlements of various sizes [42, 43] and reflects the travel time from an urban settlement to a centre with well-developed infrastructure, including banks and post offices, as well as the number of inhabitants and public services available (see [44, 45] for details). Since research indicates that residents of closely settled areas are much less willing than people in sparsely populated areas to travel to access a health care provider [17], centrality might not only reflect the type of municipality but may also be correlated with patients’ willingness to travel.

#### Regional health authorities

*Region1* through *4* are dummy variables describing whether the municipality belongs to (1) the South East, (2) West, (3) Central, or (4) North RHAs.

*Centrality0* through *3* and *Region1* through *4* are time invariant. They are part of the fixed effects and are therefore cancelled out in the model, but they are used for descriptive statistics.

#### Number of services

The dependent variable is the number of services provided at private (*Priv_Serv*), public (*Pub_Serv*), or both types of providers (*Total_Serv*) per month. This variable was calculated by accumulating claims in every municipality. For example, if a patient from municipality A goes to municipality B to receive a radiology examination, that service is classified as a service delivered to municipality A. The measurement of this variable reflects the number of services per 1000 inhabitants in the municipality.

### Hypotheses

Patients who live in the centres have better access to both public and private providers, while those who live remotely must travel up to several hours to reach a provider. The aim of the study is to investigate the interaction between patients’ travel times and the 2008 reimbursement change in terms of the number of services consumed. The Norwegian population was expected to be unevenly affected by the reimbursement change, depending on the distances to different types of the providers.

The hypotheses were based on two assumptions (A1 and A2): (A1) There is stream of patients who need services, and if one source reduces its offerings, the patients will switch to another more readily available source (both in terms of capacity and travel time); (A2) Public providers have softer budget constraints and can thus better stretch their capacity outside the limits set by budgets compared to private providers, which have hard budget constraints.

Thus, the following hypotheses were formulated.

#### Hypothesis 1

There will be a larger decrease in the number of private services than public services based on the differences in these services’ budget constraints.

#### Hypothesis 2

The stream of patients who move between providers and the effect on the total number of services will be different depending on the difference in the proximity of private and public radiology providers. The changes at private, public, and both providers will be following.

##### (2A)

Patients use private radiology more when these providers are relatively closer (i.e., *Time_difference* is negative or equal to zero), which means that, after 2008, the greatest reduction in the *Priv_Serv* will be in these areas. The reduction diminishes with the increase in *Time_difference*.

##### (2B)

The change for public providers consists of two effects. The first involves a reduction in the original public service users. The greater usage was before 2008; the greater reduction in the number of services will be after 2008. In general, patients use public radiology more when these providers are closer (that is, when *Time_difference* is zero or positive). The second effect relates to users switching from private radiology. These patients are more likely to switch the closer they live to a public provider compared to a private provider (i.e., the greater the value of *Time_difference*). Depending on what effect is greater, the change will be positive, negative, or equal to zero.

##### (2C)

Since private providers are more affected, the greatest reduction in the total number of services occurs in areas with negative *Time_difference*. This reduction will diminish with an increase in *Time_difference* because patients can more easily switch to a public provider.

**Fig. 2:**
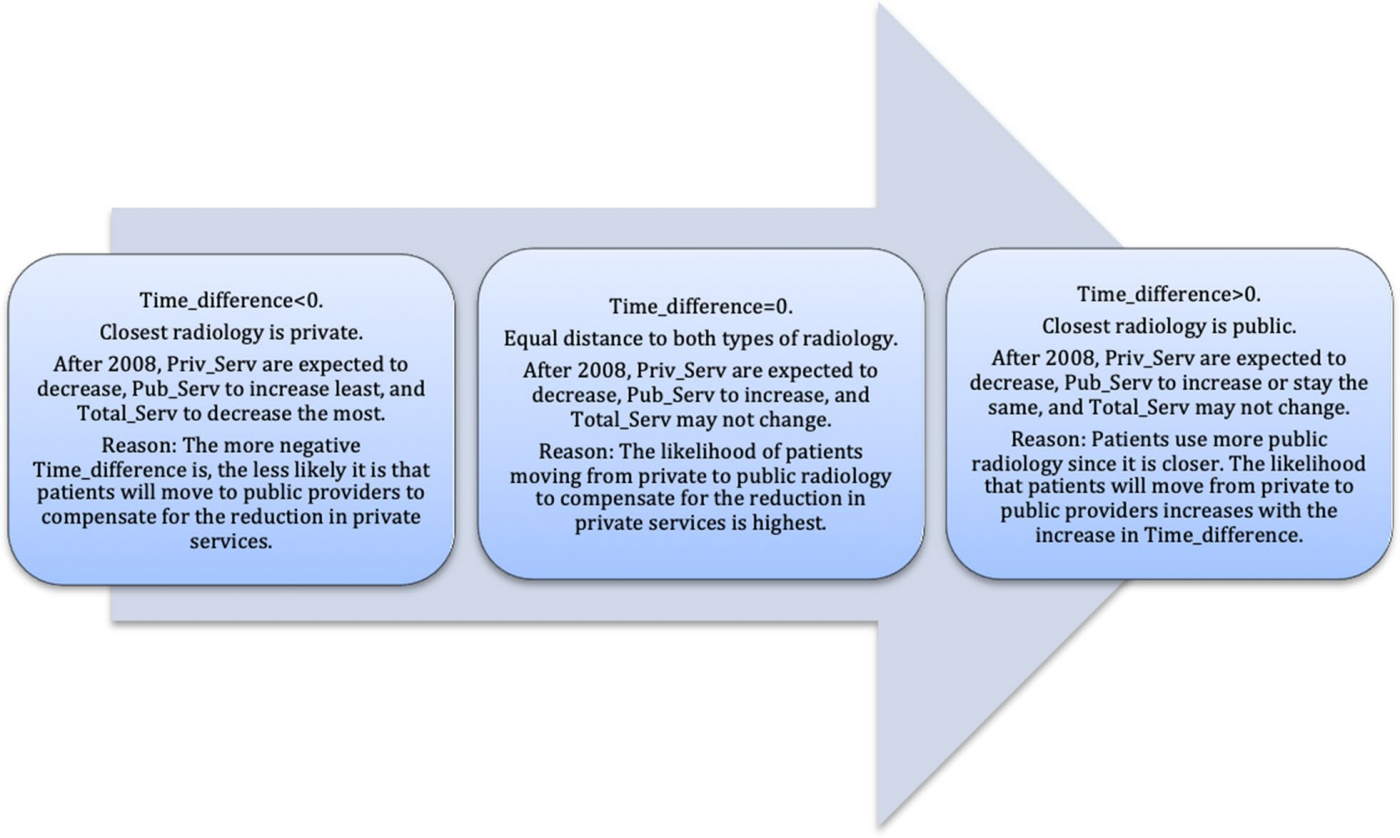
Three states on the axis of *Time_difference* (the difference in travel time between the nearest private provider and the nearest public provider) and the hypotheses regarding the consumption of radiology services

Figure 2 represents a visual explanation of the hypotheses in terms of *Time_difference*—how the consumption of services would change when moving on the scale of *Time_difference* from negative to positive values. Figure 2 makes use of three states: negative, equal to zero, and positive values of *Time_difference*. The text boxes indicate what was expected in each of the three states and why.

Following Fig. 2, the first text box indicates that the closest radiology provider is private (*Time_difference* < 0). The total number of services is expected to decrease due to reduced offerings from private providers. Since there is a longer travel time to the public provider, fewer patients would move to the public provider due to time costs compared with the other two cases (when the public provider is closer or equally close). Therefore, more patients would rather not have the radiology examination at all or have the exam out of pocket. Thus, the total number of services would decrease more than if the closest radiology provider were public.

The second box indicates that the distance between private and public radiology providers is small (*Time_difference* $\to$ 0). In this situation, patients can change providers more easily. The likelihood that patients will switch from private to public radiology provider is higher. Thus, a substantial drop in *Priv_Serv* and an increase in *Pub_Serv* is expected, while the total number of the services may not change.

In the third textbox, the closest radiology provider is public (*Time_difference* > 0). Patients are expected to use the public provider more than the private. Since public providers have softer budget constraints, the total number of services is expected to be less affected by the reimbursement change. However, some patients who used private providers before 2008 would move to public providers due to the private providers’ reduced offerings after 2008. Therefore, the total number of services is expected to stay the same, public services are expected to increase or stay the same, and private services are expected to decrease or stay the same.

### Model

A model of how *Time_difference* would affect number of services for private, public, and both providers after the reimbursement change was estimated. Time-invariant heterogeneity is controlled for without observing it through the panel data. A fixed effects model was used because it is more robust and needs fewer assumptions fulfilled than a random effects model. The fixed effects model is based on the assumption that the errors are uncorrelated with the independent variables and that the errors are conditionally homoscedastic and not serially correlated [46].

The relationship between number of the services and the *Time_difference* was not expected to be completely linear. Thus, after trying several polynomial functions, a quadratic function was chosen. A regression model was estimated separately for each of the samples of private and public providers, as well as for the sample including both types of providers:

$$\begin{aligned} {\text{Y}}_{\text{it}}& = {\text{ B}}_{0} + {\text{ B}}_{ 1} post08_{\text{t}} + {\text{ B}}_{ 2} post08_{\text{t}} \cdot Time\_difference_{\text{it}} \hfill \\ & \quad + {\text{ B}}_{ 3} post08_{\text{t}} \cdot Time\_difference_{\text{it}}^{ 2} + {\text{ e}}_{\text{i}} + {\text{ u}}_{{ 1 {\text{it}}}} \hfill \\ \end{aligned}$$

where Y_it_ denotes the number of services (*Priv_Serv*, *Pub_Serv*, *Total_Serv*) to municipality *i* (*i *= 1,…,422) in period *t* (*t *= 1,…,48), *post08*_t_ is a dummy equal to 0 prior to 2008 and 1 after 1 January 2008, and B_k_ (k = 0…3) are the regression coefficients; e_i_ is a provider specific fixed effect, and u_1it_ is an error term.

*Pubtime*_it_, *Privtime*_it_, and *Time_difference*_it_ do not vary much over time for the same municipalities, ‘it’-indexes were still used to indicate even a small variation (although the variation is not enough to keep them as independent variables in the fixed-effects model without the interaction effect with *post08*).

## Results

### Descriptive statistics

#### Travel times

Tables 1, 2, and 3 in Appendix display descriptive statistics at different levels for 4 years: before and after the change for the whole country (Table 1), before and after the change according to each level of centrality (Table 2), and before and after the change for each RHA (Table 3). Continuous variables (number of services and travel times) are described with median and interquartile range because they are not normally distributed and have many outliers, while dummy variables (centralities and RHAs) are described with means.

The median of driving time to the nearest private provider is 2 h nationally, but ranges from 20 min in *Centrality3* (largest municipalities) to 2.5 h in *Centrality0* (smallest municipalities), and from approximately 1 h in South East RHA to nearly 5.8 h in North RHA. However, there are many outliers: providers can be in the same municipality or in another region (up to 18 h away).

The median of driving time to the nearest public provider is approximately 45 min nationally. This figure ranges from 15 min in the largest municipalities to 1 h 15 min in smallest municipalities, and from 30 min in South East RHA to 1 h 45 min in North RHA. This variable had fewer outliers than travel time to private providers: in the same municipality to almost 6.5 h away.

**Fig. 3:**
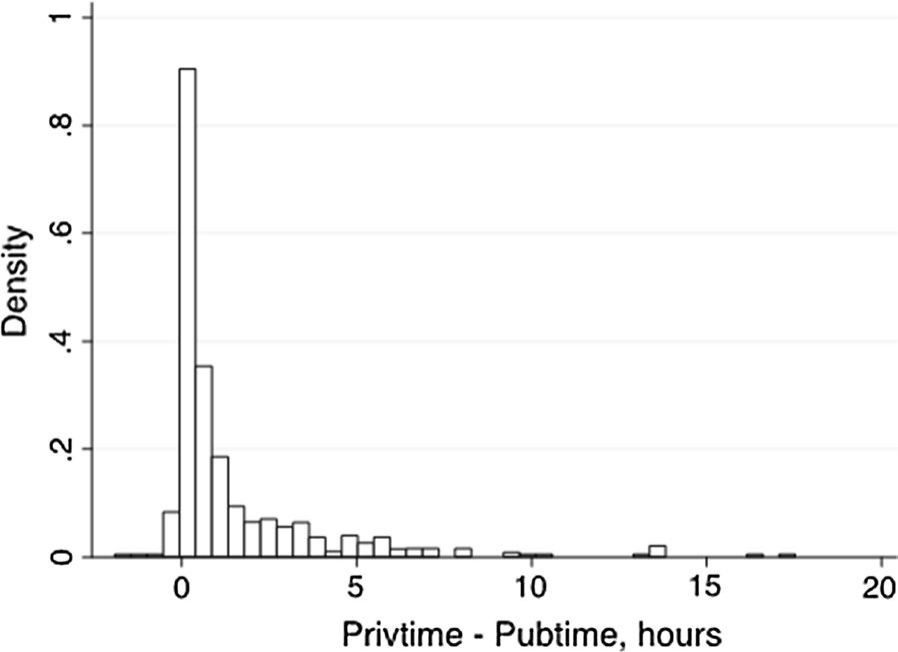
Distribution of *Time_difference* (the difference in travel time between the nearest private provider and nearest public provider) in hours

Figure 3 illustrates the distribution of the variable *Time_difference*, which is continuous, concentrated around zero, and mostly to the right-hand side of zero. The distribution has a long right tale and a left-sided truncation. There are many municipalities in which both types of providers were equally close (30%). In addition, in many municipalities, public providers were much closer than private providers (i.e., observations to the right of zero, 65%). In a few municipalities, private providers were closer than public providers (i.e., observations to the left of zero, 5%). The average *Time_difference* was about 1.5 h, although its median was 0.5 h with the 25th and 75th percentiles at 0 and 1.72 h.

#### Number of services

The summary statistics for the entire Norwegian population (Table 1) indicate that the median number of examinations per 1000 inhabitants in the municipality conducted at private providers per month has decreased from 8.22 to 6.65, while the corresponding number for public providers has increased from 23.72 to 26.04 after the change. The median of the total number did not change.

Table 2 demonstrates that the use of private providers increases with the greater centrality of municipality. The median of the number of private examinations decrease by around two to three examinations per 1000 inhabitants in the municipality, except the least central municipalities where the reduction was slightly less than 1. However, examinations at public providers increased by 3.5 per 1000 inhabitants at the least central locations and by 1.5 in the most central municipalities. In total, there was a slight increase in the numbers in the least central municipalities and a slight decrease in the most central municipalities.

Table 3, illustrating the summary statistics stratified by the RHA, demonstrates that private services (range 2 to 9 examinations/1000 inhabitants x municipality after the reform) were most strongly represented in the South East, followed by the West and Central municipalities, with the smallest number located in the North. All decreased by one to two services. Public services (range 20 to 30 examinations/1000 inhabitants x municipality after the reform) were most strongly represented in the North, followed by the Central and South East areas, with the smallest number located in the West. Public services increased by one to three services after the reform (Central, North, South East, and West, in descending order). The total number (range 29 to 36 examinations/1000 inhabitants x municipality after the reform) stayed essentially the same in the South East and the North but decreased by two in the West and increased by two in the Central RHA.

### Regressions

The models were estimated using xtreg in Stata 13. To test the first hypothesis, the coefficients next to ‘*post08*’ in Table 4 were studied. After the reimbursement change, patients received fewer services at private providers (coefficient = − 1.913) and more services at public providers (coefficient = 1.439) than before 2008. The total number of examinations has declined (coefficient = − 0.474). The descriptive statistics in Tables 1, 2, and 3 complete the picture, especially the overviews stratified by centrality and RHA.

Thus, Hypothesis 1 is supported: The number of services offered by private providers has declined more than the number of services at public providers. Furthermore, *Pub_Serv* has increased, which indicates that the stream of the patients switching from private to public providers is greater than the reduction in public services due to the 2008 reimbursement change.

**Fig. 4:**
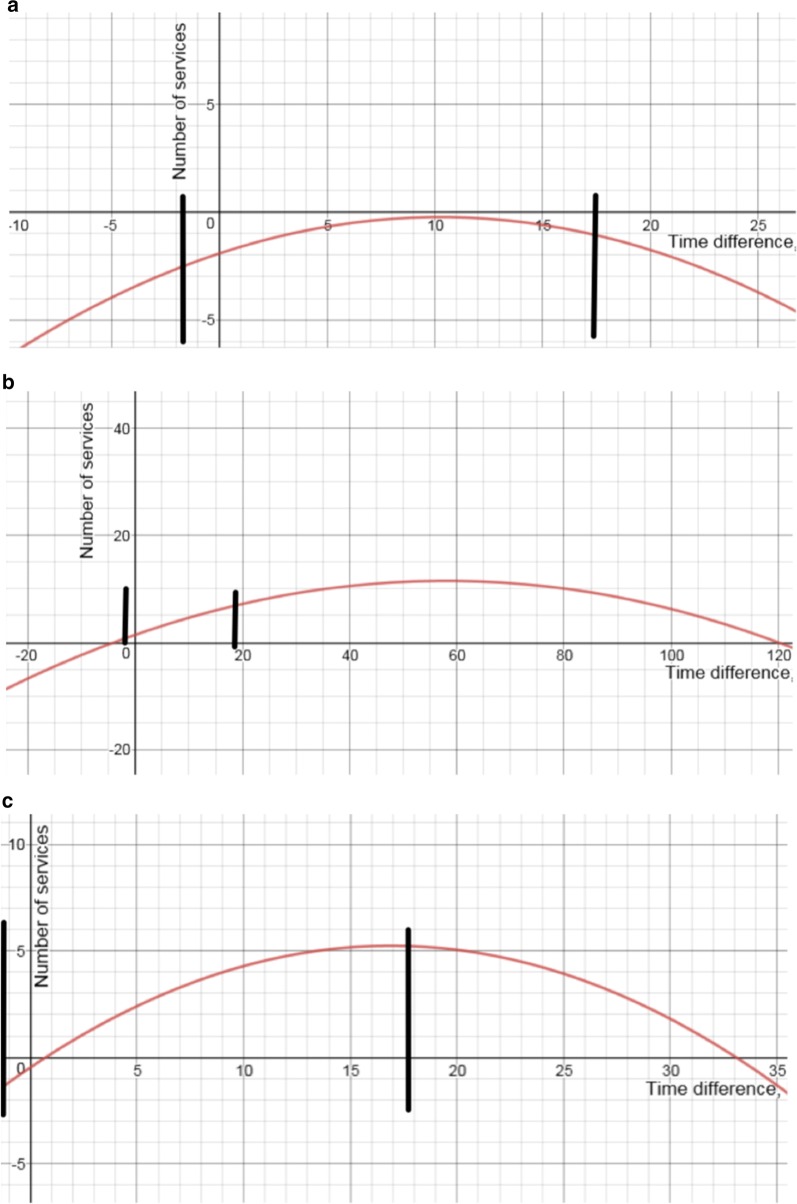
**a** Curve representing the results for the number of services at private providers and the minimum and maximum *Time_difference* (black lines). The number of services was measured in services/1000 inhabitants. The *Time_difference* was measured in hours. **b** Curve representing the results for the number of services at public providers and the minimum and maximum *Time_difference* (black lines). The number of services was measured in services/1000 inhabitants. The *Time_difference* was measured in hours. **c** Curve representing the results for the total number of services, as well as the minimum and maximum *Time_difference* (black lines). The number of services was measured in services/1000 inhabitants. The *Time_difference* was measured in hours

To test the second hypothesis, the combined coefficients for ‘*post08*’ + ‘*Time_difference* x *post08*’ + ‘*Time_difference*^2^ x *post08*’ were studied together (see Table 4). Figure 4a–c represent quadratic functions based on the coefficients in Table 4 after 2008 for easier understanding of the results for ‘private’, ‘public’, and ‘total number’ of services. All the curves are described completely; however, the study’s main interest is to examine the values within the minimum and maximum range of *Time_difference*, which are − 1.92 and 17.53 h, respectively. The curves describe the change in the number of services after 2008.

First, for private providers (Fig. 4a), the curve is below 0, indicating a reduction in services after 2008. However, the reduction diminished along *Time_difference* until it equalled 10. Then, the reduction increased again in absolute value.

Second, regarding public providers (Fig. 4b), within the minimum and maximum range of *Time_difference*, the curve is positive, increasing, and almost linear. Therefore, public services increased in conjunction with *Time_difference* in the minimum and maximum ranges.

Third, for total number of services (Fig. 4c), within the minimum and maximum range of *Time_difference*, for the values of *Time_difference* below 40 min, the curve is below the x-axis (i.e., the number of services decreases). For the values of *Time_difference* greater than 40 min, there was an increase in the services after 2008, along with *Time_difference*. If *Time_difference* equalled 40 min, then there was zero change in the number of total services after 2008.

In areas with an equal distance to both types of providers, when *Time_difference* = 0, there was a small reduction in the total number of services. Hence, the effect of the reduction in private services was stronger than the compensation in public services in the municipalities with equal distances to both types of providers.

Thus, the graphs indicate private services decreased, reducing more for negative values of *Time_difference*, with a diminishing reduction until *Time_difference* reached approximately 10 h. Public services increased along with *Time_difference*, and total services decreased until *Time*-*difference* equalled 40 min and increased for *Time_difference* values above 40 min. Hypotheses 2A, 2B, and 2C are thus supported by the results.

### Variation measure

To evaluate the results, a variation measure of the change based on the summary statistics results was included. The variation measure used the inequality measure techniques, which were adapted to this case [47–49]. The smallest and the greatest values of medians were compared before and after 2008 for different centralities and RHAs for private, public, and both services. The results are presented in Table 5a, b in Appendix.

The measure was calculated as follows. For every line for private, public, and both services in Tables 2 and 3, the smallest and the greatest values of medians for *Priv_Serv*, *Pub_Serv.* and *Total_Serv*, were chosen for before and after 2008 and transferred to Table 5a, b, respectively. Afterward, the difference between the smallest and greatest values was calculated for before and after 2008 (column ‘Difference’). The results are displayed in percentages in the column ‘Change, %’ (calculated as ‘*Difference after 2008*—*Difference before 2008’ *×* 100*%/‘*Difference before 2008’*). For the purposes of the reform evaluation, the row with total number of services is of greatest interest.

After 2008, the range for the total number of services according to centralities decreased from range [32.1–36.35] to range [33.13–35.14] (services per capita), indicating that less variation occurred between municipalities belonging to different centralities. If different RHAs are compared, variation increases. The range increased from [31.50–36.41] before 2008 to [29.63–36.49] after 2008 (services per capita).

The results reveal that the difference in the range (variation) reduced between different centralities by 53.1% and increased between different RHAs by 40.6%. However, in the Central RHA, the total number increased from 32 to 34.5, whereas in the West RHA, the number decreased from 31.5 to 29.6. These findings do not create a uniform picture of the consumption of radiology services according to location.

## Discussion

The 2008 reimbursement change affected municipalities differently, depending on the relative distance to the provider. Distance to provider has proven to play a substantial role in the health care consumption patterns, not only in Norway but internationally. Distance to the health care providers is an important factor for patients [50]. Demand for healthcare services changes amongst other factors due to variations in the travel time required to receive services, so service utilization is inversely related to travel times [12–15, 51]. Thus, the closest providers are used most frequently. Pagano et al. (2007) analysed use of radiotherapy in a Northern Eastern Italian region and found that wide geographical variation implies lack of the equity in access to services. The levels of utilization reduced greatly with increased distance from nearest radiotherapy service provider, in particular for the elderly [12]. Arcuiry et al. analysed data for regular check-ups and chronic care from North Carolina, the USA. They also conclude that there is continuing inequity in rural health care utilization, and much of it is connected to distance to a health care provider [14]. Littenberg et al. investigated the Vermont Diabetes Information System and found that adults with type 2 diabetes who live farther from their source of primary care are significantly less likely to use insulin [15]. Furthermore, a study by Cook (2018) on American data suggests that rural patients need to travel greater distance to receive health care. These patients think in terms of hours, while urban patients prefer not to travel more than 20 min to receive their health care. Thus, if rural patients view the local health care options as lacking quality or capability, they will travel longer distance to urban centres [52].

After the reimbursement change, the private providers offered fewer services, and thus patients switched to more accessible public providers. Thus, on national level there was a shift in the number of services toward public providers after the reimbursement change, which is as well supported by the report from the Norwegian Health Economics Administration [33]. The report also indicates that refunds for private and public providers were 49%/51% in 2005–2007; after 2008, they became 46%/54%. That shift might explain the results for the total number of services.

This is where the notion of relative distances becomes important. First, for lower values of *Time_difference* patients used the closest provider (in this case, a private provider) more often, and since private providers reduced their offer more after 2008, proportionally the reduction became greater for these municipalities compared to the municipalities where public providers were closer. Second, a possible transfer to a public provider is connected with time costs since public providers are farther away. Therefore, more patients fall off, (i.e. they either chose not to have certain examinations, paid out of pocket, or used private health insurance). Thus, with the increase in *Time_difference* (i.e. a public provider becomes relatively closer than a private one), the number of the public services increases because it is easier for patients to transfer to the provider that is both available and relatively closer than a private provider. Since *Time_difference* varies substantially across Norway, the geographical variation in the service provision is difficult to avoid. Several Norwegian studies also found variation in the radiology provision [22–24]. Geographical variation in health care is a problem not only in Norway but in other countries as well. For instance, an earlier study from Sweden found a significant variation in the use of radiology services between regions especially considering differences in demographics and reimbursement systems [53]. England and Belgium also face the problem of geographical variation within supply and accessibility to primary health care [54–56].

In general, the fact that in some areas many patients transferred from private to public providers is not necessarily efficient because it indicates that patients that are rationed by private providers are now treated by public providers, shifting the market from private to public providers and implying that not only patients with the lowest expected benefit were rationed from private providers. Thus, when comparing these results to Norwegian and international literature, it is difficult to fully support the statement that geographical variation in the reaction to reimbursement change seems to origin from overuse of services, although in general overuse and underuse of radiology services have shown to be a challenge in many countries including Norway [8–10, 23]. When it comes to the research of the reimbursement change in this study, the coexistence of private and public providers have a heavier role than the heterogenous use of services, as it was already pointed out in 2002 [24]. Heterogeneity of budget constraints between public and private providers, and these providers’ distribution, seems to have a greater impact in the variation among radiology services in Norway. If policy makers want a coherent effect across providers, all providers should have hard budget constraints.

### Limitations and further research

This study is missing data regarding wait times, examinations paid fully out of pocket, examinations covered by private health insurance, the number of dropped examinations, and provider capacities (i.e., the optimal workload for the providers in terms of efficiency). It would have been beneficial to obtain data for more years prior to the change to have more material with which to study the effects of the change. This limitation was accommodated by including information on radiology development for 2002–2009 from Norwegian Health Administration (Fig. 1) in the present descriptive study. In addition, control for life-expectancy, income level, and variation in education in the municipalities would have been useful. To accommodate this limitation, these characteristics were included in municipality fixed effects.

It is also important to identify the criteria used to decide whether a service is necessary. In general, all diagnostics are necessary. However, from a health economics perspective, the marginal health benefits (improvement in health) gained from services received should be measured. However, diagnostics do not constitute a procedure to improve health but rather a step to determine how to achieve better health outcomes.

Further research is required regarding whether the reduction in total services nationally was due to some of these services being unnecessary (especially in areas where patients had providers close by) or whether people stopped waiting or paying for radiology examinations privately out of pocket [16, 30, 57]. The number of patients using private health insurance increased 12 times from 2006 to 2016, and 30% of such plans were used for specialists and diagnostics [58]. Thus, investigating whether the reimbursement change added to the general equity in healthcare access would be valuable: whether it increased offers of radiology services in areas with underused services and reduced offers in areas of overuse, accounting for individual need for services [59, 60]. For regional variation for radiology services, further examination of the degree to which the overuse and underuse of the services come from GP preferences [61], patients’ specific characteristics [62–64], or purely from organizational structure [61, 65] would add clarity to the understanding of the variation in radiology services.

## Conclusion

The geographical distribution of the providers and the different ways that providers react to changes in the reimbursement system affect the implications of the reimbursement change for publicly reimbursed providers in 2008. Policymakers can take three messages from these findings. First, populations that only had private providers nearby were more affected than others by the reimbursement change in terms of the reduced number of services. Second, the reimbursement change contributed to the reallocation of patients from private to public providers. Third, the reimbursement change reduced the difference between different centralities of municipalities in their consumption pattern and increased the difference between different RHA regions.

## Data Availability

These data are sensitive without general access, so I applied for an exemption from the obligation to release the data publicly.

## Appendix

See Tables 1, 2, 3, 4 and 5.

**Table 1:** Variables and summary statistics nationally before/after the change of 1 January 2008

Variable	Explanation of variables	2007–2010		Before 2008		After 2008	
		Median	IQR	Median	IQR	Median	IQR
*Priv_Serv*	Number of services per 1000 inhabitants in the municipality done at private providers per month, services/1000 inhabitants	6.98	9.13	8.22	10.32	6.65	8.72
*Pub_Serv*	Number of services per 1000 inhabitants in the municipality done at public providers per month, services/1000 inhabitants	25.46	14.27	23.72	13.32	26.04	14.43
*Total_Serv*	Total number of services per 1000 inhabitants in the municipality per month, services/1000 inhabitants × month	33.95	12.43	33.95	12.29	33.95	12.49
*Privtime*	The travel time from the municipality of the patient^b^ to the nearest municipality with a private radiology provider in hours	1.55	2.58	1.57	2.61	1.55	2.50
*Pubtime*	The travel time from the municipality of the patient^b^ to the nearest municipality with a public radiology provider in hours	0.82	1.17	0.80	1.17	0.82	1.17
*Time_difference*	*Privtime*-*Pubtime* in hours	0.50	1.72	0.50	1.70	0.50	1.72

	Dummy variables	Mean		Mean		Mean	
*Centrality0*	Dummy for least central municipalities: Centrality 0A and 0B^a^, population less than 1000	0.33		0.34		0.33	
*Centrality1*	Dummy for Centrality 1A and 1B^a^, population from 1000 to 15,000	0.33		0.32		0.33	
*Centrality2*	Dummy for Centrality 2A and 2B^a^, population from 15,000 to 50,000	0.26		0.26		0.26	
*Centrality3*	Dummy for most central municipalities: Centrality 3^a^, population from 50,000	0.08		0.08		0.08	
*Region1*	Dummy for the Regional Health Authority South East	0.41		0.40		0.41	
*Region2*	Dummy for the Regional Health Authority West	0.20		0.20		0.20	
*Region3*	Dummy for the Regional Health Authority Central	0.19		0.19		0.19	
*Region4*	Dummy for the Regional Health Authority North	0.20		0.20		0.20	
*N* *n*		*19,867* *422*		*4944* *415* ^c^		*14,923* *422* ^c^	

“Before 2008”, the data covers 2007, or 12 periods; “After 2008”, the data covers 2008–2010, or 36 periods; IQR, interquartile range; N, number of observations; n, number of municipalities

^a^Centralities classified by Statistics Norway

^b^Municipality of the GP of the patient was used as a proxy of the municipality of the patient, as patients usually choose a GP in the same municipality, except in municipalities that are so small that the nearest GP is in another nearest municipality

^c^The difference in the number of municipalities in the data is due to structure of claims data. The total number of municipalities is 422

**Table 2:** Summary statistics stratified according to centrality before and after 1 January 2008

Variable	Centrality0				Centrality1				Centrality2				Centrality3			
	Before		After		Before		After		Before		After		Before		After	
	Median	IQR	Median	IQR	Median	IQR	Median	IQR	Median	IQR	Median	IQR	Median	IQR	Median	IQR
*Priv_Serv*	5.31	8.98	4.84	8.08	7.61	7.61	5.76	6.19	10.34	11.16	8.49	9.76	17.97	11.49	15.09	10.28
*Pub_Serv*	23.98	14.24	27.52	14.66	25.00	11.81	26.20	12.91	24.23	12.93	26.27	14.49	15.81	9.88	17.32	11.72
*Total_Serv*	32.06	13.98	33.84	14.38	33.79	12.02	33.13	12.13	35.47	11.47	35.14	11.96	36.35	8.96	34.09	9.10
*Privtime*	2.40	2.38	2.35	2.35	1.80	2.00	1.80	2.07	0.81	1.18	0.80	1.18	0.33	0.47	0.33	0.47
*Pubtime*	1.25	1.15	1.28	1.13	0.95	0.98	0.98	0.98	0.32	0.72	0.30	0.72	0.24	0.45	0.24	0.45
*Time_difference*	0.75	2.43	0.73	2.37	0.62	1.62	0.63	1.65	0.41	0.90	0.38	0.90	0.00	0.23	0.00	0.23

	Mean		Mean		Mean		Mean		Mean		Mean		Mean		Mean	
*Region1*	0.24		0.25		0.33		0.33		0.58		0.58		0.82		0.82	
*Region2*	0.23		0.24		0.27		0.26		0.11		0.11		0.06		0.06	
*Region3*	0.32		0.32		0.17		0.18		0.08		0.08		0.09		0.09	
*Region4*	0.22		0.20		0.23		0.23		0.22		0.22		0.03		0.03	
*N* *n*	1661 140		4990 145		1603 135		*4896* 137		*1272* 106		*3813* 106		408 34		1224 34	

N, number of observations; n, number of municipalities; “Before”, the data covers 2007, or 12 periods; “After”, the data covers 2008–2010, or 36 periods

**Table 3:** Summary statistics stratified according to Regional Health Authority before and after 1 January 2008

Variable	Region1				Region2				Region3				Region4			
	Before		After		Before		After		Before		After		Before		After	
	Median	IQR	Median	IQR	Median	IQR	Median	IQR	Median	IQR	Median	IQR	Median	IQR	Median	IQR
*Priv_Serv*	10.93	10.41	9.10	9.24	9.42	10.68	7.26	9.00	7.38	7.69	6.91	7.80	2.53	4.65	1.28	3.46
*Pub_Serv*	23.40	13.16	25.88	14.62	19.93	12.86	20.64	14.65	23.60	10.40	26.38	11.46	28.22	15.00	30.47	14.26
*Total_Serv*	36.41	11.71	36.49	11.84	31.53	10.91	29.63	10.48	32.15	10.36	34.53	10.65	33.18	14.51	32.96	14.03
*Privtime*	0.92	1.35	0.92	1.33	1.70	2.23	1.65	2.23	1.45	1.47	1.45	1.47	5.73	3.77	5.77	3.90
*Pubtime*	0.50	0.80	0.50	0.80	0.82	0.83	0.85	0.82	0.88	1.05	0.97	1.05	1.75	2.05	1.75	2.07
*Time_difference*	0.32	0.73	0.32	0.73	0.52	1.90	0.38	1.90	0.25	1.18	0.23	1.18	4.42	5.25	4.47	5.33

	Mean		Mean		Mean		Mean		Mean		Mean		Mean		Mean	
*Centrality0*	0.20		0.20		0.39		0.40		0.55		0.55		0.36		0.33	
*Centrality1*	0.26		0.27		0.44		0.43		0.30		0.30		0.36		0.38	
*Centrality2*	0.37		0.37		0.15		0.15		0.11		0.11		0.28		0.28	
*Centrality3*	0.17		0.17		0.02		0.02		0.04		0.04		0.02		0.01	
*N* *n*	2002 168		6078 170		982 82		2967 83		949 80		2878 82		1011 85		3000 87	

N, number of observations; n, number of municipalities; “Before”, the data covers 2007, or 12 periods; “After”, the data covers 2008–2010, or 36 periods

**Table 4:** Fixed-effects linear regression models for the number of private, public, and total services

	Private	Public	Total
*Post08*	− 1.913***	1.439***	− 0.474***
	(0.070)	(0.141)	(0.164)
*Time_difference* × *post08*	0.328***	0.348***	0.676***
	(0.049)	(0.100)	(0.116)
*Time_difference*^2^ × *post08*	− 0.016***	− 0.003	− 0.020**
	(0.004)	(0.008)	(0.010)
Cons	9.579***	24.273***	33.852***
	(0.048)	(0.097)	(0.113)
*R* ^*2*^	0.043	0.017	0.005

Coefficients (standard errors): *p < 0.10, **p < 0.05, ***p < 0.01. n = 422, N = 19,867, n = number of municipalities, N = number of obs. (n = 422; N = 19,867). *R*^*2*^= coefficient of determination. Xtreg procedure in Stata 13 was used for estimations

**Table 5:** Change in the variation among number of private, public and both services before and after 1 January 2008 (services per capita)

	Before 2008			After 2008			Change, %
	Median1	Median2	Difference	Median1	Median2	Difference	
A) Among centralities							
*Priv_Serv*	5.31	17.97	12.66	4.84	15.09	10.25	− 19
*Pub_Serv*	15.8	25	9.19	17.32	27.52	10.2	11
*Total_Serv*	32.1	36.35	4.29	33.13	35.14	2.01	− 53
B) Among Regional Health Authorities (RHAs)							
*Priv_Serv*	2.53	10.93	8.4	1.28	9.1	7.82	− 7
*Pub_Serv*	19.9	28.22	8.29	20.64	30.47	9.83	19
*Total_Serv*	31.5	36.41	4.88	29.63	36.49	6.86	41

*Median 1*—the smallest value of medians across A) Centralities, B) RHAs before 1 January 2008

*Median 2*—the highest value of medians across A) Centralities, B) RHAs after 1 January 2008

*Difference *=* Median2* *− Median1*

*Change *= (*Difference after 2008* *− Difference before 2008)* × *100*%/*Difference before 2008*

## Abbreviations

ABF: activity-based funding
CBT or CAT scan: computed body tomography
GP: general practitioner
HELFO: Norwegian Health Economics Administration
MRI: magnetic resonance imaging
NHI: National Health Insurance
RHA: Regional Health Authority
UI: ultrasound imaging
